# Salt stress alters the selectivity of mature pecan for the rhizosphere community and its associated functional traits

**DOI:** 10.3389/fpls.2025.1473473

**Published:** 2025-03-26

**Authors:** Mengting Shi, Tao Qin, Zhenyang Pu, Zhengfu Yang, Kean-Jin Lim, Menghua Yang, Zhengjia Wang

**Affiliations:** ^1^ State Key Laboratory of Subtropical Silviculture, Zhejiang A&F University, Hangzhou, Zhejiang, China; ^2^ School of Basic Medical Sciences and Forensic Medicine, Hangzhou Medical College, Hangzhou, China; ^3^ College of Animal Science and Technology, College of Veterinary Medicine, Zhejiang A & F University, Key Laboratory of Applied Technology on Green-Eco Healthy Animal Husbandry of Zhejiang Province, Zhejiang Provincial Engineering Laboratory for Animal Health Inspection and Internet Technology, Hangzhou, Zhejiang, China

**Keywords:** mature pecan, rhizosphere, microbiome, selectivity, salt stress

## Abstract

**Introduction:**

Salt stress is a major global environmental factor limiting plant growth. Rhizosphere bacteria, recruited from bulk soil, play a pivotal role in enhancing salt stress resistance in herbaceous and crop species. However, whether the rhizosphere bacterial community of a mature tree can respond to salt stress, particularly in saline-alkalitolerant trees, remains unexplored. Pecan (*Carya illinoinensis*), an important commercially cultivated nut tree, is considered saline-alkali tolerant.

**Methods:**

Pecan trees (12 years) were subjected to different NaCl concentrations for 12 weeks. Collected samples included bulk soil, rhizosphere soil, roots, leaves, and fruit. Amplicon sequencing data and shotgun metagenomic sequencing data obtained from the samples were investigated: 1) microbial communities in various ecological niches of mature pecan trees; 2) the characteristic of the rhizosphere bacteria community and the associated functional traits when pecan suffered from salt stress.

**Results and discussion:**

We characterized the mature pecan-associated microbiome (i.e., fruit, leaf, root, and rhizosphere soil) for the first time. These findings suggest that niche-based processes, such as habitat selection, drive bacterial and fungal community assembly in pecan tissues. Salt stress reduced bacterial diversity, altered community composition, and shifted pecan’s selective pressure on *Proteobacteria* and *Actinobacteria*. Shotgun metagenomic sequencing further revealed functional traits of the rhizosphere microbiome in response to salt stress. This study enhances our understanding of mature tree-associated microbiomes and supports the theory that shaping the rhizosphere microbiome may be a strategy for saline-alkali-tolerant mature trees to resist salt stress. These findings provide insights into salt tolerance in mature trees and suggest potential applications, such as the development of bio-inoculants, for managing saline environments in agricultural and ecological contexts.

## Introduction

Plants provide diverse niches for microbial growth and proliferation, forming complex associations that enhance plant productivity and health in natural environments (Trivedi et al., 2020). The interaction between plants and their microbiota creates a “holobiont”, in which evolutionary selection between plants and microorganisms contributes to system stability (Vandenkoornhuyse et al., 2015). High-throughput sequencing has significantly advanced our understanding of plant-associated microorganisms (Bulgarelli et al., 2015; Hamonts et al., 2018; Zhang et al., 2022; Xiong et al., 2021; Bettenfeld et al., 2022), yet research on microbial communities associated with mature trees remains limited to species such as citrus and poplar (Xu et al., 2018; Timm et al., 2018).

Salt stress is a major global challenge that limits plant growth and crop productivity, exacerbated by poor irrigation, population pressure, and industrial pollution (Sahab et al., 2021). To adapt to salt stress, plants have developed various strategies to integrate exogenous salinity stress signals with endogenous developmental cues, optimizing the balance between growth and stress responses. These strategies include the formation of salt-excreting glands or trichomes, reestablishing cellular ionic, osmotic, and reactive oxygen species equilibrium, and regulating key developmental processes such as flowering time (Yang and Guo, 2018; Kazan and Lyons, 2016). A particularly promising approach involves plant growth-promoting bacteria (Jalili et al., 2009; Bharti et al., 2016; Dong et al., 2019). For example, *Sphingomonas* and *Microcoleus* enhance the salt tolerance in groundnut (Xu et al., 2020), while *Bacillus* spp. and *Pseudomonas* spp. improve salt tolerance in various crops, including maize, barley, rice, wheat, tomato, chickpea, cotton, soybean, cucumber, peanut, sunflower, and oats (Xie et al., 2024). Furthermore, Abdelfadil et al. (2024) revealed the influence of salinity on bacterial microbiome assembly in halophytes and crops. Moreover, recent studies have reported conflicting findings on the effects of salinity stress on the complexity and stability of microbial communities. Environmental stressors generally reduce microbial community complexity and stability (Hernandez et al., 2021). Guan et al. (2021) found that increased soil salinity significantly reduced the complexity of bacterial communities. However, Ji et al. (2019) reported that microbial communities exposed to salinity stress exhibited greater complexity than those in nonsaline conditions. These different findings highlight the complexity of microbial responses to salinity stress. While most research has focused on herbaceous plants and crops, few studies have investigated whether salt stress induces changes in the rhizosphere microbiome of mature trees at the community level, particularly saline-alkali-tolerant species.

The establishment of microbial communities in the rhizosphere is not random but is strongly influenced by host plant selection (Bulgarelli et al., 2013; Agler et al., 2016). Plants have the capacity to modify soil microbiota by secreting specific molecules into the rhizosphere, a phenomenon known as the “rhizosphere effect”. These plant-secreted molecules play a crucial role in shaping the rhizosphere microbiome (Stringlis et al., 2018; Hu et al., 2018). Since abiotic stresses, such as salt stress, can alter the composition of root exudates (Chai and Schachtman, 2022), it is plausible that changes in root exudates also influence the composition and structure of rhizosphere microbial communities. This possibility requires further investigation.

Pecan (*Carya illinoinensis*), a saline-alkali-tolerant tree from the genus *Carya* (family *Juglandaceae*) (Huang et al., 2019), is a commercially significant nut tree valued for its nutritional and health benefits. However, the microbial communities associated with different compartments of mature pecan plants, as well as the response of the rhizosphere bacterial community and its functional traits to salt stress, remain unexplored. This study aims to investigate microbial communities across various ecological niches of mature pecan trees and to characterize the rhizosphere bacterial community and its functional traits under salt stress.

## Materials and methods

### Sample collection

Twelve-year-old pecan trees were grown at Zhejiang Agriculture and Forestry University (30.25°N, 119.72°E), Hangzhou, China. The trees were subjected to different NaCl concentrations (0.3% (w/v) NaCl and 0.6% (w/v) NaCl) for 12 weeks. Salt irrigation was applied to the entire surrounding area, with each tree receiving 5 L of saline water every week until 12 weeks. In total, 27 samples were obtained, as listed in **Supplementary Table S1**.

Collected samples included bulk soil, rhizosphere soil, roots, leaves, and fruit. Root samples were taken from a depth of 20 cm below ground level. Rhizosphere soil was strictly defined as soil particles adhering to the roots, while bulk soil was sampled from the same depth interval at the edge of the excavation hole. Leaf samples were obtained from terminal (mature) leaves along multiple branches. For fruit samples, four fruits were collected from each tree.

### Processing of samples

The samples were processed in our laboratory following the method described by Beckers et al. (2017) and ours, with the following modifications (Shi et al., 2023). In brief, bulk soil samples were processed by removing plant debris, placed in sterile bags, and stored at − 80°C. Root samples were pooled and washed three times with 10 mM PBS (130 mM NaCl, 7 mM Na_2_HPO_4_, 3 mM NaH_2_PO_4_, pH 7.4). The washed-off soil was poured into a 50-mL centrifuge tube, centrifuged, and stored at − 80°C. Root, leaf, and fruit compartments were first rinsed with sterile ddH_2_O for 30 s, then sequentially treated with 70% (v/v) ethanol for 2 min and sodium hypochlorite (2.5% sodium hypochlorite with 0.1% Tween 80) for 5 min. The tissues were further washed with 70% (v/v) ethanol for 30 s to remove epiphytic bacteria and fungi, then rinsed five times with sterile ddH_2_O. After sterilization, plant samples were homogenized under aseptic conditions. Finally, quadruple aliquots (1.5 mL) of homogenized plant samples (roots, leaves, and fruits) from each sample were stored at − 80°C until DNA extraction.

### DNA extraction and sequencing

DNA was extracted from soil samples using the Qiagen PowerSoil DNA Extraction Kit (QIAGEN Strasse 1, 40724 Hilden, Germany) and from plant samples using the Invisorb Spin Plant Mini Kit (Stratec Biomedical AG, Birkenfeld, Germany), both following the manufacturer’s protocols. DNA quality and quantity were determined using a Nanodrop spectrophotometer (Thermo Scientific, Wilmington, DE, USA). Extracted DNA samples were stored at − 80°C.

For amplicon library preparation, the amplification of 16S DNA fragments was performed using common amplified primers and methods for prokaryotic rRNA genes. Distinct regions (16S V4) were amplified using a specific primer (16S V4: 515F-806R) with a barcode. Sequence libraries were generated using TruSeq^®^ DNA PCR-Free Sample Preparation Kit (Illumina, University of California, San Diego, USA) following the manufacturer’s recommendations, and index codes were added. The library quality was assessed using the Qubit@ 2.0 Fluorometer (Thermo Scientific) and an Agilent Bioanalyzer 2100 system. The library was sequenced on the Illumina NovaSeq platform, generating 250 bp paired-end reads.

For metagenomic library preparation, extracted DNA was fragmented to an average size of approximately 400 bp using Covaris M220 (Covaris, Massachusetts, USA) for paired-end library construction. The paired-end library was constructed using NEXTFLEX Rapid DNA-Seq Kit (Bioo Scientific, Austin, USA). Adapters containing the full complement of sequencing primer hybridization sites were ligated to the blunt ends of the fragments. Paired-end sequencing was performed using an Illumina HiSeq (Illumina Inc, USA) at Majorbio in Shanghai, China.

### Amplicon analysis

The OTUs in each subgroup were considered present only if they appeared in at least 60% of the samples. Subsequent analyses were conducted based on these data. Alpha diversity was used to analyze the complexity and diversity of the samples. All these indices were calculated and displayed using the amplicon package in R software (version 4.2.0). The statistical significance of the alpha diversity index was tested using ANOVA and Tukey-HSD. PCA based on Bray–Curtis was applied to compare microbiome communities between groups. The different OTUs between groups were calculated using EdgeR in the “edgeR” R package at significance thresholds of *p* cutoff < 0.05 and FDR < 0.05 and were presented as Volcano plots. The genera present in all samples were regarded as nodes and were used to calculate the correlations among them to simulate the network structure of the microbial communities. Nodes with *R* > 0.9 and *p* < 0.01 were selected and visualized with Gephi.

### Metagenomic data analysis

The data were analyzed on the online Majorbio cloud platform (http://www.majorbio.com). Briefly, the paired-end Illumina reads were trimmed of adaptors, and low-quality reads (length < 50 bp, quality value < 20, or containing N bases) were removed using Fastp (50) (https://github.com/OpenGene/fastp; version 0.20.0). Metagenomics data were assembled using MEGAHIT (Li et al., 2015) (https://github.com/voutcn/megahit; version 1.1.2), which utilizes succinct de Bruijn graphs. Contigs with a length of ≥ 300 bp were selected as the final assembling result, and these contigs were then used for gene prediction and annotation. Open reading frames (ORFs) from each assembled contig were predicted using Prodigal (Hyatt et al., 2010) (https://github.com/voutcn/megahit; v1.1.2). The predicted ORFs with a length of ≥ 100 bp were retrieved and translated into amino acid sequences using the NCBI translation table (http://www.ncbi.nlm.nih.gov/Taxonomy/taxonomyhome.html/index.cgi?chapter=tgencodes#SG1). Nonredundant gene catalogs were constructed using CD-HIT (Fu et al., 2012) (http://www.bioinformatics.org/cd-hit/; version 4.6.1) with 90% sequence identity and 90% coverage. High-quality reads were aligned to the nonredundant gene catalogs to calculate gene abundance with 95% identity using SOAPaligner (Li et al., 2008) (https://github.com/ShujiaHuang/SOAPaligner; version soap 2.21). The taxonomic annotation was performed based on the nonredundant gene set. Representative sequences of the nonredundant gene catalog were aligned to the nonredundant (NR) database with an *E*-value cutoff of 1*e*−5 using Diamond (Buchfink et al., 2015) (https://github.com/bbuchfink/diamond) for taxonomic annotations. To obtain functional information for the unigenes, the protein sequences were homology searched against the KO database using Diamond software with an *E*-value cutoff of 1*e*−5. The annotation of the COGs of proteins for the ORFs was performed using BLASTP against the egg-NOG database (v4.5) with an *E*-value cutoff of 1*e*−5. ORFs from the metagenome were analyzed using the KEGG and COG databases to identify functional profiles.

### Comparative analysis

We used the DESeq2 method to statistically assess differences in the abundance profiles of KEGG level 3 and NOGs between nonsalt- and salt-treated conditions. The data were analyzed using the online tool of the Majorbio Cloud Platform (https://cloud.majorbio.com/page/tools/).

## Results

### Microbial community composition shifts across pecan habitats

**Figure 1:**
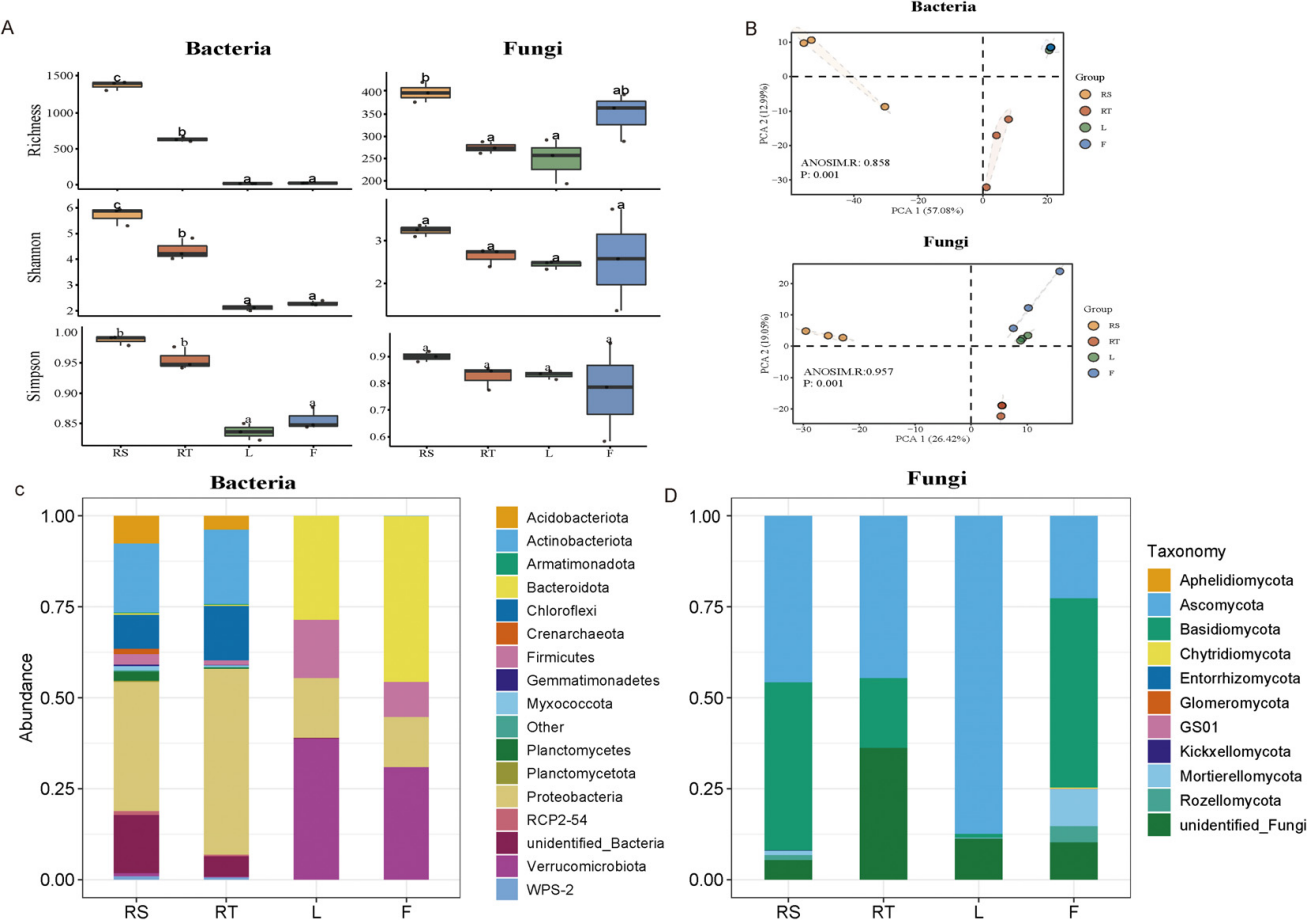
Composition and diversity of microbiota in different ecological niches of mature pecan. **(A)** Alpha diversity (index: Richness, Shannon, Simpson) across broad habitats (habitats: the location where microorganisms are found in plants: rhizosphere soil, root, leaf, and fruit) for both bacterial and fungal communities. Box plots show the high, low, and median values, with the lower and upper edges of each box representing the first and third quartiles, respectively. The *x*-axis represents the sample information. Statistical significance between different groups is indicated by different letters (*p* < 0.05, ANOVA). **(B)** Beta diversity across broad habitats (rhizosphere soil, root, leaf, and fruit) for both bacterial and fungal communities. Beta diversity is visualized using principal component analysis (PCA) based on the unweighted UniFrac distance at the operational taxonomic unit (OTU) level. The variation explained by the plotted principal coordinates is indicated in the axis labels. *p*-values from Adonis tests, adjusted by the FDR, are displayed at the top of each PCA plot. **(C, D)** Structure of bacterial and fungi communities across broad habitats (rhizosphere soil, root, leaf, and fruit). The relative abundances of bacterial **(C)** and fungi **(D)** communities are shown at the phylum level. RS, rhizosphere soil; RT, root; L, leaf; F, fruit.

To investigate the diversity of the mature pecan-associated microbiome, we analyzed alpha diversity across four major pecan habitats: fruit, leaf, root, and rhizosphere soil. Our results showed that the alpha diversity of bacteria was highest in soil habitats and lowest in leaf habitats (**Figure 1A**). Leaf and fruit exhibited similar levels of bacterial alpha diversity. For fungi, alpha diversity was higher in fruit than in leaf or root habitats and was also higher in soil than in roots (**Figure 1A**). We performed principal component analysis (PCA) using the Bray–Curtis algorithm to assess community composition among the four habitats. The PCA results indicated that samples clustered into three distinct groups: root and rhizosphere soil formed separate clusters, while leaf and fruit grouped together (**Figure 1B**). These findings suggest that belowground niches exert a strong influence on microbial community composition. At the phylum level, amplicon analysis revealed that *Proteobacteria* and *Actinobacteria* dominated the belowground niches, whereas *Verrucomicrobiota* and *Proteobacteria* were most abundant in aboveground niches (**Figure 1C**). For fungi, *Ascomycota* and *Basidiomycota* were the dominant phyla across all four habitats (**Figure 1D**). The significant differences in microbial diversity and community composition across the four pecan habitats highlight the influence of habitat-specific conditions on microbial community structure.

### Effects of salt stress on the α-diversity and network complexity of pecan rhizosphere bacteria

**Figure 2:**
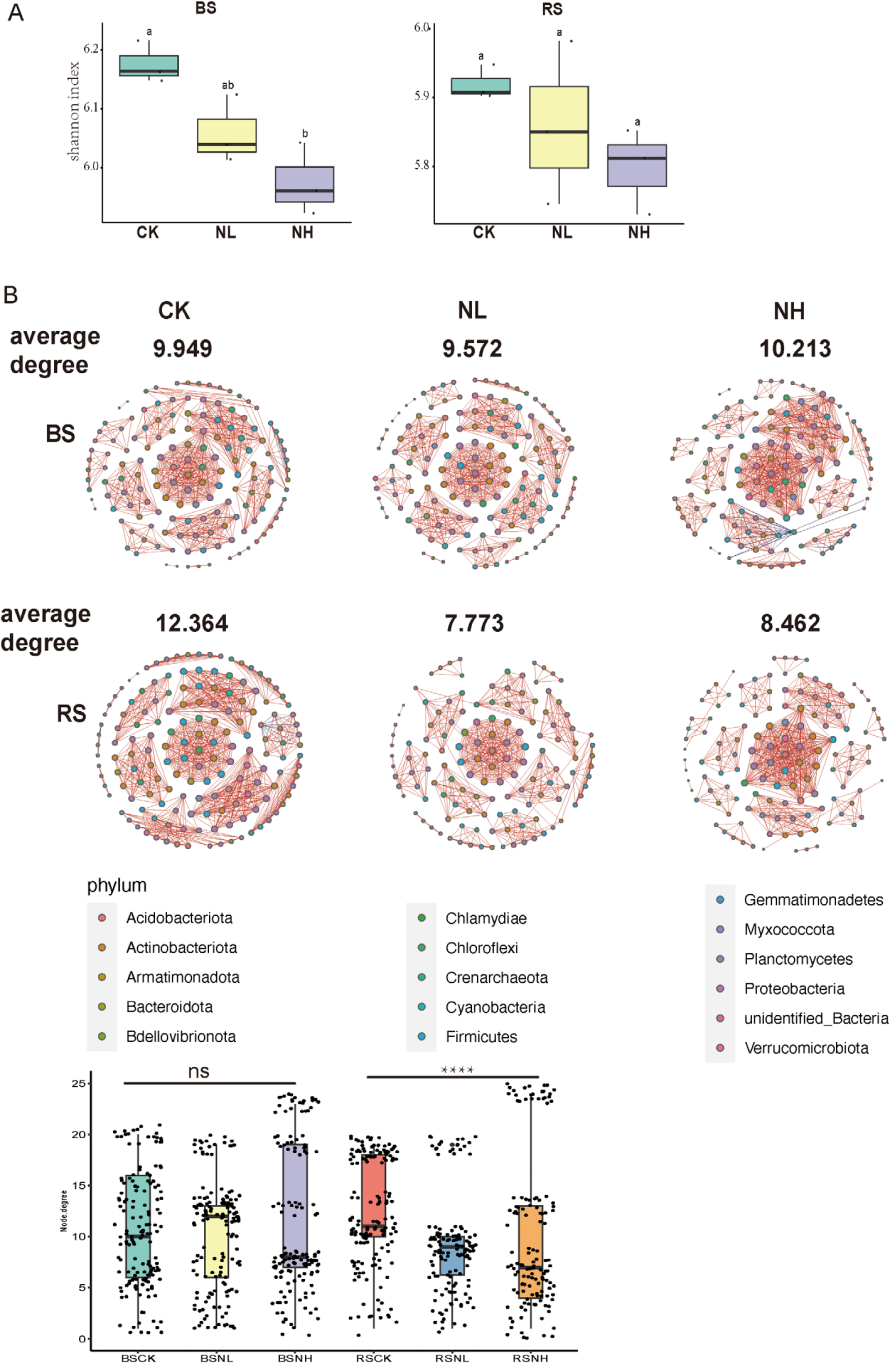
Effects of salt stress on the α-diversity and network complexity of rhizosphere bacteria in pecan. **(A)** Alpha diversity of bulk soil (left) and rhizosphere soil (right) under nonsalt-, low-salt-, and high-salt-treated conditions for bacterial communities, based on amplicon sequencing data. Box plots show the high, low, and median values, with the lower and upper edges of each box representing the first and third quartiles, respectively. BS, bulk soil; RS, rhizosphere soil; CK, control check (nonsalt condition); NL, low concentration (0.3%) NaCl; NH, High concentration (0.6%) NaCl; BSCK, bulk soil under nonsalt condition; BSNL, bulk soil under low-salt condition; BSNH, bulk soil under high-salt condition; RSCK, rhizosphere soil under nonsalt condition; RSNL, rhizosphere soil under low-salt condition; RSNH, rhizosphere soil under high-salt condition. **(B)** Network co-occurrence analysis of bacterial communities in bulk soil and rhizosphere soil samples under nonsalt-, low-salt-, and high-salt-treated conditions. (Spearman |*ρ*| > 0.7 and *p* < 0.05). Each node represents the taxonomic level of the genus (based on 16S rRNA), with red lines indicating positive correlations and blue lines indicating negative correlations. Line width represents the strength of the correlation, while node colors denote different phyla. Box plots show the node size of each genus in the samples. ^****^ *p* < 0.0001. ns, non significant.

Salt stress influences the composition and activity of root exudates, thereby altering rhizosphere microbial diversity and structure to enhance plant resistance to stress. To assess its impact on the α-diversity of rhizosphere bacteria, we compared the Shannon index among nonsalt-, low-salt-, and high-salt-treated groups for both bulk soil and rhizosphere soil microbiomes. The results showed a decline in bacterial α-diversity with increasing salt concentration in both bulk and rhizosphere soil samples, indicating that salt stress reduces bacterial diversity (**Figure 2A**; **Supplementary Figure S1**).

To further investigate the impact of salt stress, we analyzed the network complexity of rhizosphere bacteria under different salt concentrations. Network complexity, measured by the average degree of the network, decreased from 12.364 in nonsalt-treated groups to 8.402 in high-salt-treated groups within the rhizosphere, whereas bulk soil bacteria exhibited no significant changes (**Figure 2B**). These findings indicate that salt stress not only reduces the α-diversity but also diminishes the network complexity of rhizosphere bacteria, potentially affecting microbial interactions and functions.

### Salt stress induces the variation of the community composition of pecan rhizosphere bacteria

**Figure 3:**
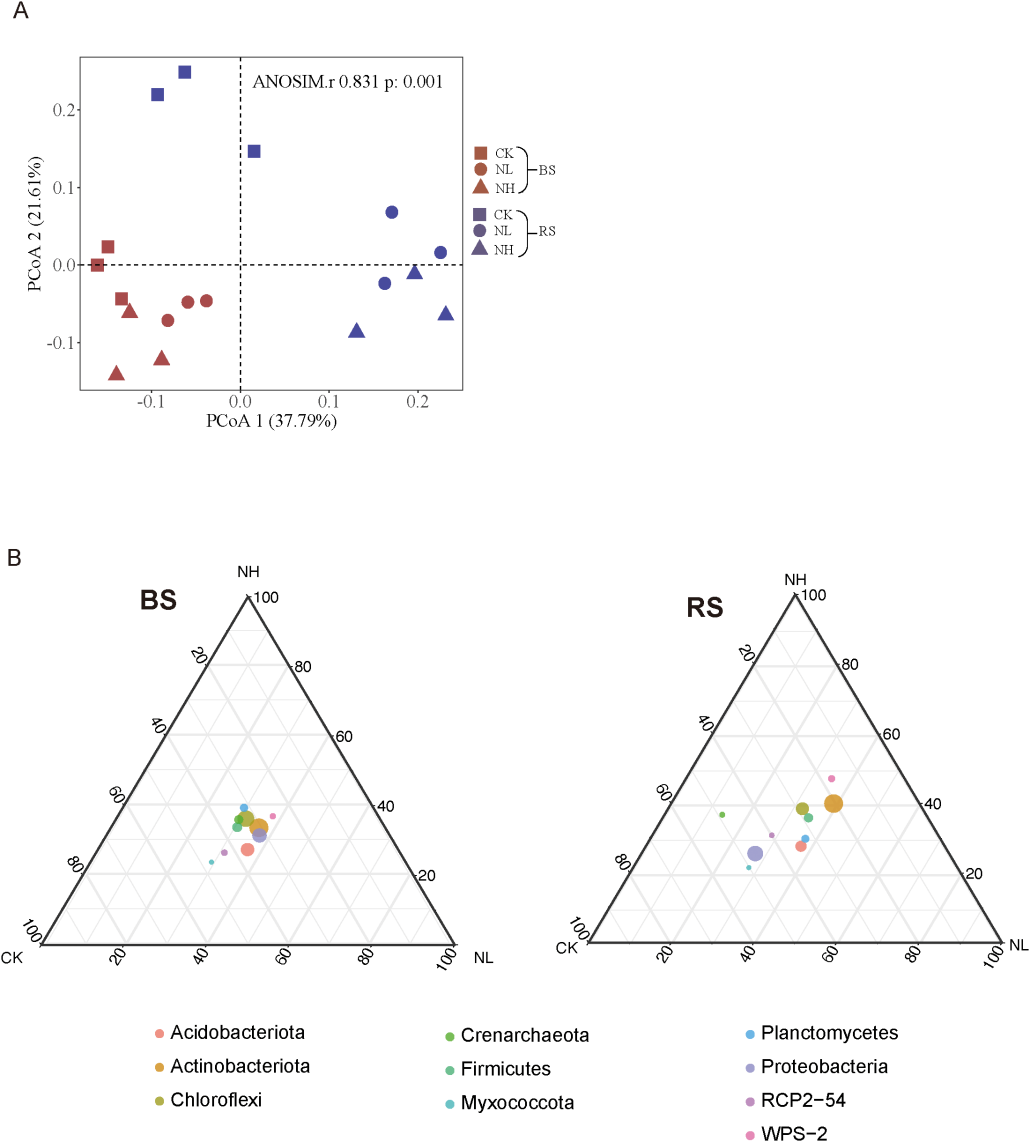
Salt stress alters the community composition of pecan rhizosphere bacteria. **(A)** Beta diversity is visualized using principal coordinates analysis (PCoA) based on Bray–Curtis distance at the operational taxonomic unit (OTU) level. **(B)** Ternary plots illustrating bacterial community composition in bulk soil (left) and rhizosphere soil (right).

Principal coordinate analysis (PCoA) was performed to assess the impact of salt stress on bacterial community composition. The results showed clear differences between nonsalt- and salt-treated rhizosphere groups, whereas the corresponding bulk soil groups exhibited overlapping patterns (**Figure 3A**). This suggests that pecan actively modulates its rhizosphere bacterial community in response to salt stress.

To examine the rhizosphere effect—where microorganisms are attracted to nutrients exuded by plant roots (Bais et al., 2006)—dominant microbial phyla were visualized in a ternary plot under different salt concentrations for bulk and rhizosphere soil samples (**Figure 3B**). In the plot, different salt concentrations were positioned at the three vertices, and the dot size represented the relative abundance of microbes in each group. While microbial communities in bulk soil remained relatively dense, rhizosphere communities exhibited increased dispersion and irregularity under salt stress. These results indicate that salt stress induces significant changes in the bacterial community within the rhizosphere niche. Collectively, our findings highlight the profound impact of salt stress on the diversity, structure, and complexity of rhizosphere microbes in mature pecan (e.g., α-diversity, β-diversity, and network complexity).

### Salt stress switched the selectivity of pecan on Proteobacteria and Actinobacteria

**Figure 4:**
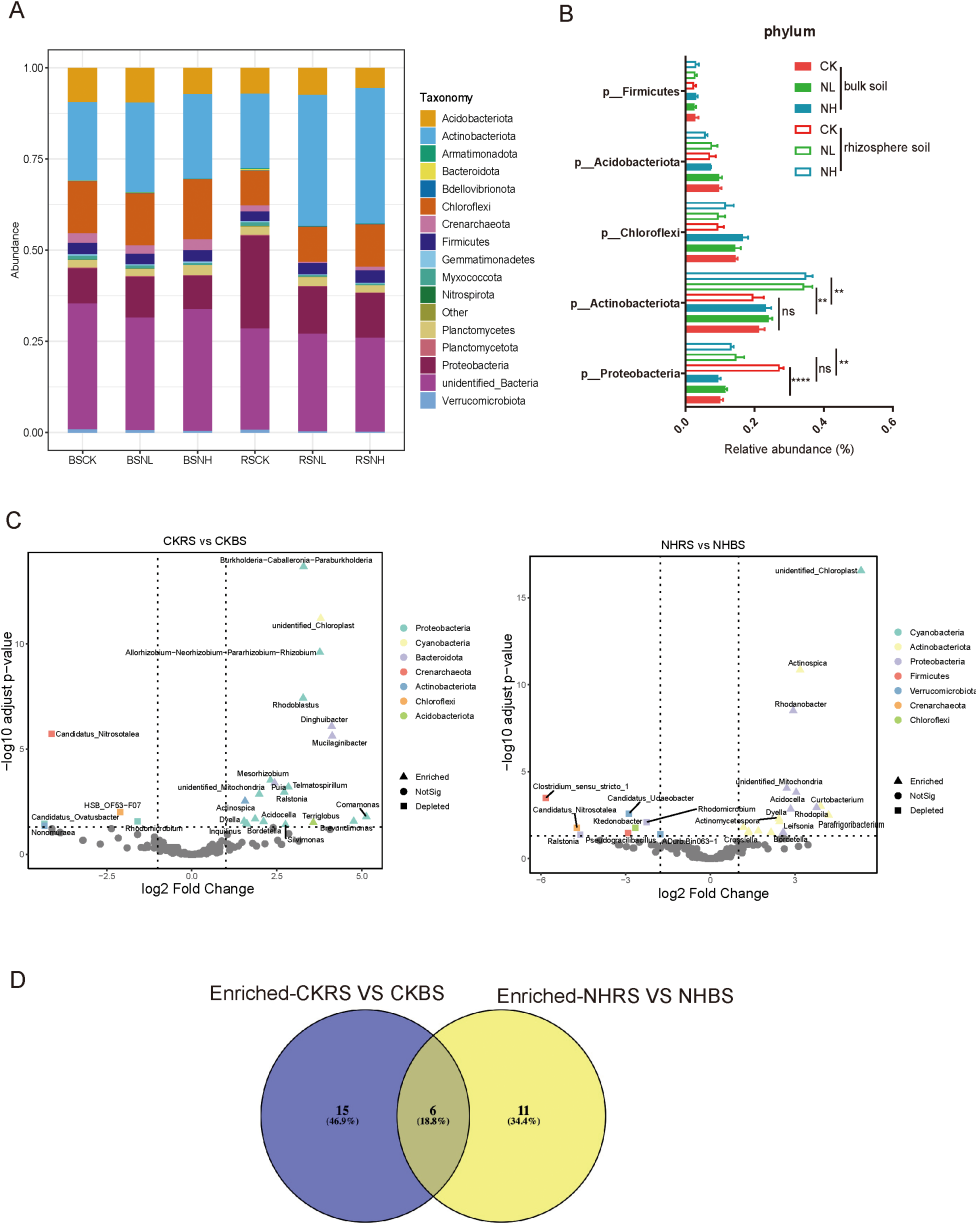
Salt stress altered the selectivity of pecan toward *Proteobacteria* and *Actinobacteria*. **(A)** Structure of the bacterial community in bulk soil and rhizosphere soil samples under nonsalt-, low-salt-, and high-salt-treated conditions. BSCK, bulk soil under nonsalt condition; BSNL, bulk soil under low-salt condition; BSNH, bulk soil under high-salt condition; RSCK, rhizosphere soil under nonsalt condition; RSNL, rhizosphere soil under low-salt condition; RSNH, rhizosphere soil under high-salt condition. **(B)** Average relative abundance of the five most abundant genera in samples based on 16S data. **(C)** Volcano plots showing enrichment and depletion analyses for CKRS versus CKBS and NHRS versus NHBS comparisons. CKBS, bulk soil under nonsalt condition; CKRS, rhizosphere soil under nonsalt condition; NHBS, bulk soil under high-salt condition; NHRS, rhizosphere soil under high-salt condition. The shape of each dot represents the enrichment and depleted taxonomic affiliation of the OTUs at the genus level, with colors indicating phylum-level annotations. **(D)** Venn diagram illustrating the unique and shared genera predicted from the enriched genera datasets of CKRS versus CKBS and NHRS versus NHBS comparisons.

Rhizosphere microbiomes, primarily derived from bulk soil, play a crucial role in plant resistance to abiotic stress. Amplicon sequencing of pecan rhizosphere samples identified *Proteobacteria*, *Actinobacteria*, *Chloroflexi*, *Acidobacteriota*, and *Firmicutes* as the dominant phyla (**Figure 4A**). To determine whether salt stress affects pecan’s selectivity for these phyla, their relative abundances were compared between bulk soil and rhizosphere soil across different salt treatments (nonsalt, low-salt, and high-salt conditions). Under salt stress, the abundance of *Actinobacteria* significantly increased in the rhizosphere compared to bulk soil, whereas no such difference was observed in nonsalt-treated conditions, indicating enhanced selectivity for *Actinobacteria* under salt stress. Conversely, the abundance of *Proteobacteria* in the rhizosphere was significantly lower than in bulk soil under normal conditions, but this selectivity disappeared under salt stress (**Figure 4B**). At the genus level, enrichment and depletion analyses were conducted on rhizosphere and bulk soil groups under nonsalt and salt-stress conditions (**Figure 4C**). Under nonsalt conditions, 21 genera were enriched in the pecan rhizosphere, while 17 genera were enriched under salt stress (**Supplementary Data Sheet S1**). A Venn diagram of these enriched genera revealed that 11 were specifically recruited under salt stress, with *Conexibacteria* being the most abundant bacteria (**Figure 4D**; **Supplementary Data Sheet S1**).

### Effects of salt stress on rhizosphere microbiome functions

**Figure 5:**
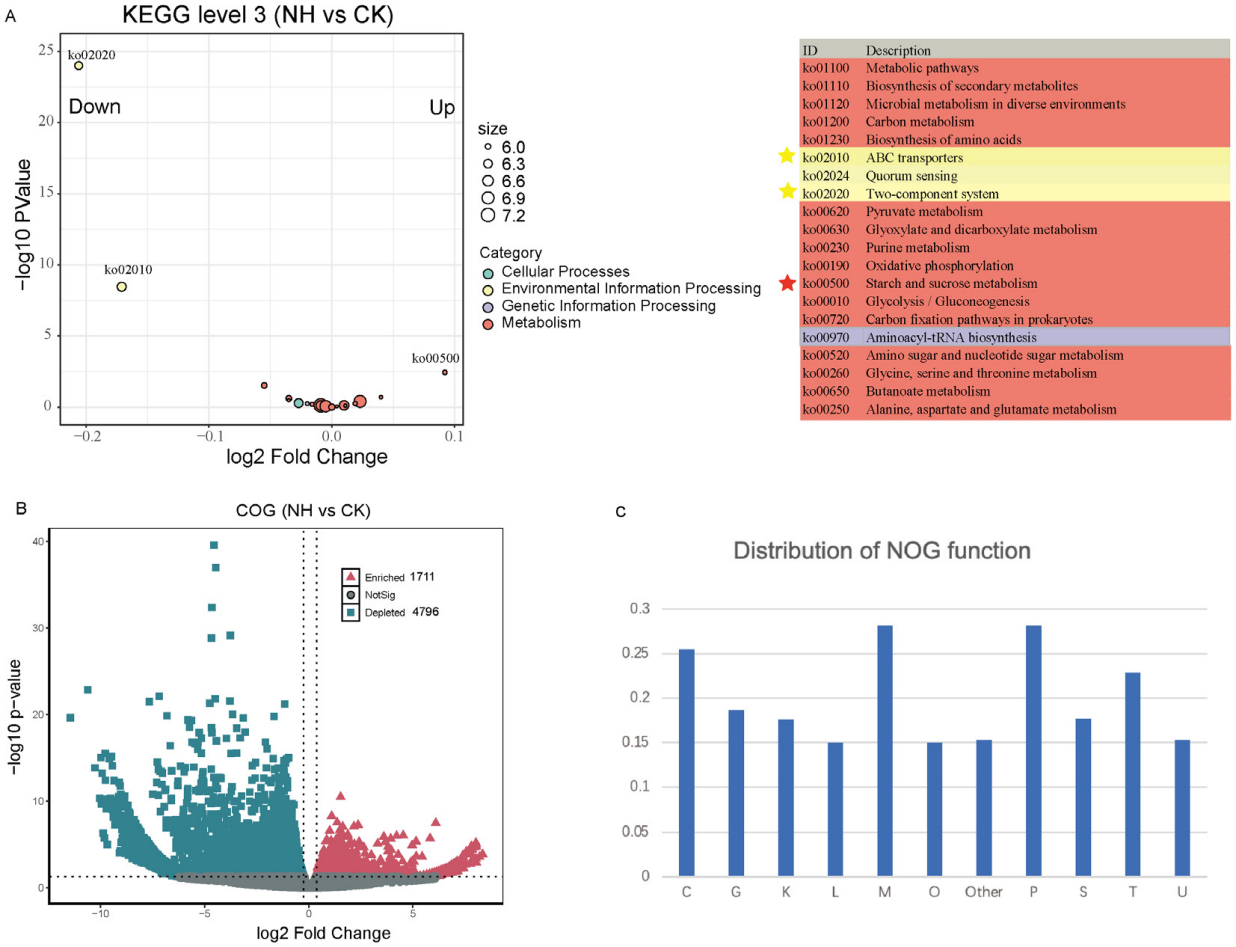
Effects of salt stress on rhizosphere microbiome functions. **(A)** The top 20 KEGG pathways show significant differences in abundance between the nonsalt- and high-salt-treated groups. Each dot is color-coded according to KEGG level 1 annotation. **(B)** Volcano plots depicting enrichment and depletion analyses between the nonsalt- and high-salt-treated groups. Enriched NOGs are represented by red triangles, depleted NOGs by blue squares, and nonsignificant differences by gray dots. **(C)** Distribution of different NOGs across functional categories. C, energy production and conversion; G, carbohydrate transport and metabolism; K, transcription; L, replication, recombination, and repair; M, cell wall/membrane/envelope biogenesis; O, Posttranslational modification, protein turnover, chaperones; P, inorganic ion transport and metabolism; S, function unknown; T, signal transduction mechanisms; U, intracellular trafficking, secretion, and vesicular transport.

To explore the potential functions of rhizosphere microbiota in mitigating the effects of salt stress on mature pecan, we conducted shotgun metagenome analysis under nonsalt and salt-treated conditions. Functional annotations revealed that approximately 36% of the unigenes aligned with the KEGG Orthology (KO) database and approximately 76% aligned with the Non supervised Orthologous groups (NOG) database (**Supplementary Data Sheet S2**), indicating that the combined use of the Kyoto Encyclopedia of Genes and Genomes (KEGG) and Clusters of Orthologous Groups (COG) databases enables a comprehensive analysis of functional genes in pecan rhizosphere microbial communities. Analysis of KEGG level 3 pathways showed significant enrichment of Ko00500 (starch and sucrose metabolism) under salt-treated conditions, while Ko02020 (two-component system) and Ko02010 (ABC transporters) were significantly depleted (**Figure 5A**). This suggests that these functional traits in the rhizosphere microbiome respond to salt stress. At the COG level, we compared the abundance of COGs between nonsalt- and salt-treated samples, finding that 1,711 COGs were enriched and 4,796 were depleted under salt stress (**Figure 5B**; **Supplementary Data Sheet S3**). The distribution of the enriched and depleted COG functional categories indicated that genes involved in inorganic ion transport and metabolism (28.2%) and cell wall/membrane/envelope biogenesis (28.1%) were significantly affected by salt stress (**Figure 5C**). These findings highlight the strong impact of salt stress on the functional traits of the pecan rhizosphere microbiome, particularly in pathways related to nutrient transport and structural integrity.

## Discussion

The plant-associated microbial community, often referred to as the plant’s “second genome”, plays a critical role in plant productivity. In recent years, plant–microbiome associations have received increasing attention for their role in promoting beneficial interactions that enhance agricultural sustainability. The plant niche significantly influences microbiome composition, with distinct microbial communities colonizing different plant compartments (Pereira et al., 2023; Trivedi et al., 2020; Bulgarelli et al., 2013). This study investigated microbial communities in various ecological niches of mature pecan trees and revealed significant differences in bacterial and fungal community composition (β-diversity) between above- and belowground habitats (e.g., root, leaf, soil). Bacterial α-diversity decreased across the four habitats, whereas fungal α-diversity remained unchanged (**Figure 1**). However, amplification biases (i.e., rarefying bacterial data at 500 sequences) may limit conclusions regarding microbial diversity and warrant further validation. Overall, our findings suggest that niche-based processes, such as habitat selection, drive bacterial and fungal community assembly in pecan tissues. These results align with the theoretical framework of host selection and niche occupation in mature tree microbiome assembly.

Plants are sessile and must develop suitable mechanisms to adapt to high-salt environments. Salt stress influences the composition and activity of root exudates, thereby altering rhizosphere microbial diversity and structure. The rhizosphere microbiota is crucial for enhancing plant adaptation and productivity, playing a key role in resistance to both biotic and abiotic stresses. Central to this interaction is the rhizosphere soil, which serves as a primary reservoir of microorganisms. However, research on the variability of rhizosphere microbial communities in mature trees under salt stress remains limited. Our data demonstrate that salt stress significantly impacts rhizosphere bacterial communities in mature pecan trees, reducing α-diversity, decreasing network complexity, and altering community composition (**Figures 2**, **3**). Notably, network complexity declined in the rhizosphere but remained unchanged in bulk soil under salt stress (**Figure 2B**). Similarly, salt stress induced significant variation in rhizosphere bacterial composition, whereas no such phenomenon was observed in bulk soil samples (**Figure 3A**). Network analysis offers a powerful approach to understanding microbial interactions and niche dynamics (Kajihara and Hynson, 2024; Duran-Pinedo et al., 2011; Emerson and Gillespie, 2008; Zhou et al., 2011). Environmental factors such as pH, nutrient availability, and hydrological characteristics are known to reshape ecological networks (Tylianakis et al., 2007; Barberan et al., 2012). For example, by modulating root exudate composition, plants can modify soil properties, thereby recruiting microbes that support plant survival under adverse conditions (Vives-Peris et al., 2020). Our findings suggest that salt stress modifies root exudation in mature pecan, simplifying network complexity and driving community shifts in the rhizosphere, while bulk soil remains unaffected. Prior studies have linked root exudate profiles with microbial recruitment and plant stress tolerance, including salt tolerance in ryegrass and shifts in rhizosphere bacteria in *Populus* (He et al., 2022; Cao et al., 2024). In mature pecan, salt stress appears to alter root exudates, promoting the recruitment of beneficial bacteria such as *Conexibacteria*, which may enhance salt resistance. These insights highlight the importance of rhizosphere microbiota in plant resilience and suggest potential avenues for developing biofertilizers to improve salt tolerance in sensitive crops.

Plants consistently recruit stress-resistance-promoting microbiomes from bulk soil under abiotic stress conditions, playing a crucial role in plant resistance (Fitzpatrick et al., 2019; Xu et al., 2018; Liu et al., 2021; Wu et al., 2023; Xiao et al., 2022; Timm et al., 2018). *Proteobacteria* and *Actinobacteria* are the most abundant phyla in the rhizosphere of most plants (Xu et al., 2018; Ling et al., 2022; Wang et al., 2019). In this study, we found that pecan exhibited weaker selective pressure on actinobacteria under normal conditions (nonsalt-treated condition), whereas this selectivity significantly increased under salt stress (**Figure 4B**). Consequently, *Actinobacteria* became the dominant phylum in the rhizosphere under salt-treated conditions. *Actinobacteria* are ideal candidates for promoting plant growth due to their high rhizosphere abundance, ability to colonize plant roots and surfaces, and capacity to produce plant hormones, iron carriers, secondary metabolites, and antibiotics (Narsing Rao et al., 2022; Hamedi and Mohammadipanah, 2015). Previous studies suggest that the relative abundance of microbial phyla can serve as an indicator of soil nutrient dynamics (Smit et al., 2001). Our data indicate that salt stress likely alters root exudate composition, leading to shifts in the relative abundance of *Proteobacteria* and *Actinobacteria* in the pecan rhizosphere. This shift may represent an adaptive strategy for pecan to cope with salt stress.

What functional traits of the microbiome significantly influence the rhizosphere community of mature pecans under salt stress? Our analysis revealed that genes associated with starch and sucrose metabolism (Ko00500) were significantly enriched in the pecan rhizosphere, whereas genes linked to the two-component system were significantly depleted (**Figure 5**). Carbohydrate metabolism plays a decisive factor in salt stress tolerance, as demonstrated in prior research (Thalmann et al., 2016). Our results, along with the work of others, suggest that the starch and sucrose metabolism pathway not only directly supports plant resistance to salt stress but also plays a key role in rhizosphere bacterial survival under these conditions. Conversely, the depletion of genes involved in the two-component system suggests a different adaptive mechanism (**Figure 5**). Two-component systems enable bacteria to respond to environmental stimuli and play key roles in both inter- and intramicrobial communication among different species (Zschiedrich et al., 2016; Trivedi et al., 2020). Under salt stress, the harsh rhizosphere environment may favor bacteria with specific two-component systems that enhance survival and adaptation, aiding community assembly and supporting pecan productivity. This highlights the possibility that salt stress-induced changes in root exudates, particularly carbon resources, play a pivotal role in shaping the rhizosphere microbiome community through two-component signaling. These findings provide insight into the functional shifts in the rhizosphere microbiome that enhance pecan fitness under adverse conditions.

## Conclusion

In this study, we analyzed the pecan-associated microbiome, focusing on changes in community composition and functional gene profiles in the rhizosphere under salt stress. Our findings indicate that salt stress significantly impacts the diversity and structure of rhizosphere microbes in mature pecan, altering the plant’s selective pressure on dominant bacterial populations. Additionally, the functional traits of rhizosphere bacteria, particularly their ability to perceive and utilize root exudates, were crucial for adaptation to salt stress. For the first time, we present a detailed characterization of the mature pecan-associated microbiome, highlighting key bacterial community features and functional gene traits in the rhizosphere under salt stress. These findings offer valuable insights into the strategies mature pecan trees employ to withstand salt stress and lay the foundation for enhancing stress tolerance in agricultural systems.

## Data Availability

The names of the repository/repositories and accession number(s) can be found at: https://www.ncbi.nlm.nih.gov/, PRJNA916384 and PRJNA916643.

## Supplementary material

Supplementary Table 1 Sample distribution.

Supplementary Data Sheet 1 The enriched, depleted and core genus in the pecan rhizosphere under salt stress condition.

Supplementary Data Sheet 2 Functional annotations were obtained for the proportion of unigenes by blasting against the KEGG Orthology database and the NOG database.

Supplementary Data Sheet 3 The enriched and depleted COG genes of the bacteria in the pecan rhizosphere under salt stress condition.

Supplementary Figure 1 Alpha diversity of the rhizosphere soil under nonsalt, low-salt and high-salt treated conditions for bacterial communities by using the metagenonic sequencing data. Box plots show high, low, and median values, with the lower and upper edges of each box denoting the first and third quartiles, respectively. RSCK: rhizosphere soil under nonsalt condition; RSNL: rhizosphere soil under low-salt condition; RSNH: rhizosphere soil under high-salt condition.

## References

[B1] Abdelfadil M. R. Patz S. Kolb S. Ruppel S. (2024). Unveiling the influence of salinity on bacterial microbiome assembly of halophytes and crops. Environ. Microbiome 19, 49. doi: 10.1186/s40793-024-00592-3 39026296 PMC11256479

[B2] Agler M. T. Ruhe J. Kroll S. Morhenn C. Kim S. T. Weigel D.. (2016). Microbial hub taxa link host and abiotic factors to plant microbiome variation. PloS Biol. 14, e1002352. doi: 10.1371/journal.pbio.1002352 26788878 PMC4720289

[B3] Bais H. P. Weir T. L. Perry L. G. Gilroy S. Vivanco J. M. (2006). The role of root exudates in rhizosphere interactions with plants and other organisms. Annu. Rev. Plant Biol. 57, 233–266. doi: 10.1146/annurev.arplant.57.032905.105159 16669762

[B4] Barberan A. Bates S. T. Casamayor E. O. Fierer N. (2012). Using network analysis to explore co-occurrence patterns in soil microbial communities. ISME J. 6, 343–351. doi: 10.1038/ismej.2011.119 21900968 PMC3260507

[B5] Beckers B. Op De Beeck M. Weyens N. Boerjan W. Vangronsveld J. (2017). Structural variability and niche differentiation in the rhizosphere and endosphere bacterial microbiome of field-grown poplar trees. Microbiome 5, 25. doi: 10.1186/s40168-017-0241-2 28231859 PMC5324219

[B6] Bettenfeld P. Canals I. Cadena J. Jacquens L. Fernandez O. Fontaine F.. (2022). The microbiota of the grapevine holobiont: A key component of plant health. J. Adv. Res. 40, 1–15. doi: 10.1016/j.jare.2021.12.008 36100319 PMC9481934

[B7] Bharti N. Pandey S. S. Barnawal D. Patel V. K. Kalra A. (2016). Plant growth promoting rhizobacteria Dietzia natronolimnaea modulates the expression of stress responsive genes providing protection of wheat from salinity stress. Sci. Rep. 6, 34768. doi: 10.1038/srep34768 27708387 PMC5052518

[B8] Buchfink B. Xie C. Huson D. H. (2015). Fast and sensitive protein alignment using DIAMOND. Nat. Methods 12, 59–60. doi: 10.1038/nmeth.3176 25402007

[B9] Bulgarelli D. Garrido-Oter R. Munch P. C. Weiman A. Droge J. Pan Y.. (2015). Structure and function of the bacterial root microbiota in wild and domesticated barley. Cell Host Microbe 17, 392–403. doi: 10.1016/j.chom.2015.01.011 25732064 PMC4362959

[B10] Bulgarelli D. Schlaeppi K. Spaepen S. Ver Loren van Themaat E. Schulze-Lefert P. (2013). Structure and functions of the bacterial microbiota of plants. Annu. Rev. Plant Biol. 64, 807–838. doi: 10.1146/annurev-arplant-050312-120106 23373698

[B11] Cao Y. H. Zhao X. W. Nie G. Wang Z. Y. Song X. Zhang M. X.. (2024). The salt-tolerance of perennial ryegrass is linked with root exudate profiles and microflora recruitment. Sci. Total Environ. 916, 170205. doi: 10.1016/j.scitotenv.2024.170205 38272075

[B12] Chai Y. N. Schachtman D. P. (2022). Root exudates impact plant performance under abiotic stress. Trends Plant Sci. 27, 80–91. doi: 10.1016/j.tplants.2021.08.003 34481715

[B13] Dong Z. Y. Narsing Rao M. P. Wang H. F. Fang B. Z. Liu Y. H. Li L.. (2019). Transcriptomic analysis of two endophytes involved in enhancing salt stress ability of Arabidopsis thaliana. Sci. Total Environ. 686, 107–117. doi: 10.1016/j.scitotenv.2019.05.483 31176810

[B14] Duran-Pinedo A. E. Paster B. Teles R. Frias-Lopez J. (2011). Correlation network analysis applied to complex biofilm communities. PloS One 6, e28438. doi: 10.1371/journal.pone.0028438 22163302 PMC3233593

[B15] Emerson B. C. Gillespie R. G. (2008). Phylogenetic analysis of community assembly and structure over space and time. Trends Ecol. Evol. 23, 619–630. doi: 10.1016/j.tree.2008.07.005 18823678

[B16] Fitzpatrick C. R. Mustafa Z. Viliunas J. (2019). Soil microbes alter plant fitness under competition and drought. J. Evol. Biol. 32, 438–450. doi: 10.1111/jeb.2019.32.issue-5 30739360

[B17] Fu L. Niu B. Zhu Z. Wu S. Li W. (2012). CD-HIT: accelerated for clustering the next-generation sequencing data. Bioinformatics 28, 3150–3152. doi: 10.1093/bioinformatics/bts565 23060610 PMC3516142

[B18] Guan Y. Jiang N. Wu Y. Yang Z. Bello A. Yang W. (2021). Disentangling the role of salinity–sodicity in shaping soil microbiome along a natural saline–sodic gradient. Sci. Total Environ. 765, 142738. doi: 10.1016/j.scitotenv.2020.142738 33097264

[B19] Hamedi J. Mohammadipanah F. (2015). Biotechnological application and taxonomical distribution of plant growth promoting actinobacteria. J. Ind. Microbiol. Biotechnol. 42, 157–171. doi: 10.1007/s10295-014-1537-x 25410828

[B20] Hamonts K. Trivedi P. Garg A. Janitz C. Grinyer J. Holford P.. (2018). Field study reveals core plant microbiota and relative importance of their drivers. Environ. Microbiol. 20, 124–140. doi: 10.1111/emi.2018.20.issue-1 29266641

[B21] He Y. Zhu Z. Zhou Z. Lu T. Kumar A. Xia Z. (2022). Foliar application of lambda-cyhalothrin modulates root exudate profile and the rhizosphere bacteria community of dioecious Populus cathayana. Environ. pollut. 313, 120123. doi: 10.1016/j.envpol.2022.120123 36087893

[B22] Hernandez D. J. David A. S. Menges E. S. Searcy C. A. Afkhami M. E. (2021). Environmental stress destabilizes microbial networks. ISME J. 15, 1722–1734. doi: 10.1038/s41396-020-00882-x 33452480 PMC8163744

[B23] Hu L. Robert C. A. M. Cadot S. Zhang X. Ye M. Li B.. (2018). Root exudate metabolites drive plant-soil feedbacks on growth and defense by shaping the rhizosphere microbiota. Nat. Commun. 9, 2738. doi: 10.1038/s41467-018-05122-7 30013066 PMC6048113

[B24] Huang Y. Xiao L. Zhang Z. Zhang R. Wang Z. Huang C.. (2019). The genomes of pecan and Chinese hickory provide insights into Carya evolution and nut nutrition. Gigascience 8. doi: 10.1093/gigascience/giz036 PMC6497033 31049561

[B25] Hyatt D. Chen G. L. Locascio P. F. Land M. L. Larimer F. W. Hauser L. J. (2010). Prodigal: prokaryotic gene recognition and translation initiation site identification. BMC Bioinf. 11, 119. doi: 10.1186/1471-2105-11-119 PMC2848648 20211023

[B26] Jalili F. Khavazi K. Pazira E. Nejati A. Rahmani H. A. Sadaghiani H. R.. (2009). Isolation and characterization of ACC deaminase-producing fluorescent pseudomonads, to alleviate salinity stress on canola (Brassica napus L.) growth. J. Plant Physiol. 166, 667–674. doi: 10.1016/j.jplph.2008.08.004 18829132

[B27] Ji M. Kong W. Yue L. Wang J. Deng Y. Zhu L. (2019). Salinity reduces bacterial diversity, but increases network complexity in Tibetan Plateau lakes. FEMS Microbiol. Ecol. 95, fiz190. doi: 10.1093/femsec/fiz190 31778180

[B28] Kajihara K. T. Hynson N. A. (2024). Networks as tools for defining emergent properties of microbiomes and their stability. Microbiome 12, 184. doi: 10.1186/s40168-024-01868-z 39342398 PMC11439251

[B29] Kazan K. Lyons R. (2016). The link between flowering time and stress tolerance. J. Exp. Bot. 67, 47–60. doi: 10.1093/jxb/erv441 26428061

[B30] Li D. Liu C. M. Luo R. Sadakane K. Lam T. W. (2015). MEGAHIT: an ultra-fast single-node solution for large and complex metagenomics assembly via succinct de Bruijn graph. Bioinformatics 31, 1674–1676. doi: 10.1093/bioinformatics/btv033 25609793

[B31] Li R. Li Y. Kristiansen K. Wang J. (2008). SOAP: short oligonucleotide alignment program. Bioinformatics 24, 713–714. doi: 10.1093/bioinformatics/btn025 18227114

[B32] Ling N. Wang T. Kuzyakov Y. (2022). Rhizosphere bacteriome structure and functions. Nat. Commun. 13, 836. doi: 10.1038/s41467-022-28448-9 35149704 PMC8837802

[B33] Liu T. Y. Ye N. Wang X. Das D. Tan Y. You X.. (2021). Drought stress and plant ecotype drive microbiome recruitment in switchgrass rhizosheath. J. Integr. Plant Biol. 63, 1753–1774. doi: 10.1111/jipb.v63.10 34288433

[B34] Narsing Rao M. P. Lohmaneeratana K. Bunyoo C. Thamchaipenet A. (2022). Actinobacteria-plant interactions in alleviating abiotic stress. Plants (Basel) 11. doi: 10.3390/plants11212976. PMC9658302 36365429

[B35] Pereira L. B. Thomazella D. P. T. Teixeira P. J. P. L. (2023). Plant-microbiome crosstalk and disease development. Curr. Opin. Plant Biol. 72, 102351. doi: 10.1016/j.pbi.2023.102351 36848753

[B36] Sahab S. Suhani I. Srivastava V. Chauhan P. S. Singh R. P. Prasad V. (2021). Potential risk assessment of soil salinity to agroecosystem sustainability: Current status and management strategies. Sci. Total Environ. 764, 144164. doi: 10.1016/j.scitotenv.2020.144164 33385648

[B37] Shi M. Qin T. Cheng Z. Zheng D. Pu Z. Yang Z.. (2023). Exploring the core bacteria and functional traits in pecan (Carya illinoinensis) rhizosphere. Microbiol. Spectr. 11, e0011023. doi: 10.1128/spectrum.00110-23 37310220 PMC10433825

[B38] Smit E. Leeflang P. Gommans S. van den Broek J. van Mil S. Wernars K. (2001). Diversity and seasonal fluctuations of the dominant members of the bacterial soil community in a wheat field as determined by cultivation and molecular methods. Appl. Environ. Microbiol. 67, 2284–2291. doi: 10.1128/AEM.67.5.2284-2291.2001 11319113 PMC92868

[B39] Stringlis I. A. Yu K. Feussner K. de Jonge R. Van Bentum S. Van Verk M. C.. (2018). MYB72-dependent coumarin exudation shapes root microbiome assembly to promote plant health. Proc. Natl. Acad. Sci. U. S. A. 115, E5213–E5E22. doi: 10.1073/pnas.1722335115 29686086 PMC5984513

[B40] Thalmann M. Pazmino D. Seung D. Horrer D. Nigro A. Meier T.. (2016). Regulation of leaf starch degradation by abscisic acid is important for osmotic stress tolerance in plants. Plant Cell 28, 1860–1878. doi: 10.1105/tpc.16.00143 27436713 PMC5006701

[B41] Timm C. M. Carter K. R. Carrell A. A. Jun S. R. Jawdy S. S. Velez J. M.. (2018). Abiotic stresses shift belowground populus-associated bacteria toward a core stress microbiome. mSystems 3. doi: 10.1128/msystems.00070-17 PMC5781258 29404422

[B42] Trivedi P. Leach J. E. Tringe S. G. Sa T. Singh B. K. (2020). Plant-microbiome interactions: from community assembly to plant health. Nat. Rev. Microbiol. 18, 607–621. doi: 10.1038/s41579-020-0412-1 32788714

[B43] Tylianakis J. M. Tscharntke T. Lewis O. T. (2007). Habitat modification alters the structure of tropical host-parasitoid food webs. Nature 445, 202–205. doi: 10.1038/nature05429 17215842

[B44] Vandenkoornhuyse P. Quaiser A. Duhamel M. Le Van A. Dufresne A. (2015). The importance of the microbiome of the plant holobiont. New Phytol. 206, 1196–1206. doi: 10.1111/nph.2015.206.issue-4 25655016

[B45] Vives-Peris V. de Ollas C. Gomez-Cadenas A. Perez-Clemente R. M. (2020). Root exudates: from plant to rhizosphere and beyond. Plant Cell Rep. 39, 3–17. doi: 10.1007/s00299-019-02447-5 31346716

[B46] Wang L. Li Z. Liu R. Li L. Wang W. (2019). Bacterial diversity in soybean rhizosphere soil at seedling and mature stages. Pol. J. Microbiol. 68, 281–284. doi: 10.33073/pjm-2019-023 31250597 PMC7256853

[B47] Wu S. Wu K. Shi L. Sun X. Tan Q. Hu C. (2023). Recruitment of specific microbes through exudates affects cadmium activation and accumulation in Brassica napus. J. Hazard Mater. 442, 130066. doi: 10.1016/j.jhazmat.2022.130066 36193614

[B48] Xiao X. Wang J. L. Li J. J. Li X. L. Dai X. J. Shen R. F.. (2022). Distinct patterns of rhizosphere microbiota associated with rice genotypes differing in aluminum tolerance in an acid sulfate soil. Front. Microbiol. 13, 933722. doi: 10.3389/fmicb.2022.933722 35783428 PMC9247542

[B49] Xie X. Gan L. Wang C. He T. (2024). Salt-tolerant plant growth-promoting bacteria as a versatile tool for combating salt stress in crop plants. Arch. Microbiol. 206, 341. doi: 10.1007/s00203-024-04071-8 38967784

[B50] Xiong C. Zhu Y. G. Wang J. T. Singh B. Han L. L. Shen J. P.. (2021). Host selection shapes crop microbiome assembly and network complexity. New Phytol. 229, 1091–1104. doi: 10.1111/nph.v229.2 32852792

[B51] Xu L. Naylor D. Dong Z. Simmons T. Pierroz G. Hixson K. K.. (2018). Drought delays development of the sorghum root microbiome and enriches for monoderm bacteria. Proc. Natl. Acad. Sci. U.S.A. 115, E4284–E4E93. doi: 10.1073/pnas.1717308115 29666229 PMC5939072

[B52] Xu Y. Zhang G. Ding H. Ci D. Dai L. Zhang Z. (2020). Influence of salt stress on the rhizosphere soil bacterial community structure and growth performance of groundnut (Arachis hypogaea L.). Int. Microbiol. 23, 453–465. doi: 10.1007/s10123-020-00118-0 31933013

[B53] Xu J. Zhang Y. Zhang P. Trivedi P. Riera N. Wang Y.. (2018). The structure and function of the global citrus rhizosphere microbiome. Nat. Commun. 9, 4894. doi: 10.1038/s41467-018-07343-2 30459421 PMC6244077

[B54] Yang Y. Guo Y. (2018). Elucidating the molecular mechanisms mediating plant salt-stress responses. New Phytol. 217, 523–539. doi: 10.1111/nph.2018.217.issue-2 29205383

[B55] Zhang X. Ma Y. N. Wang X. Liao K. He S. Zhao X.. (2022). Dynamics of rice microbiomes reveal core vertically transmitted seed endophytes. Microbiome 10, 216. doi: 10.1186/s40168-022-01422-9 36482381 PMC9733015

[B56] Zhou J. Deng Y. Luo F. He Z. Yang Y. (2011). Phylogenetic molecular ecological network of soil microbial communities in response to elevated CO_2_ . mBio 2. doi: 10.1128/mBio.00122-11 PMC3143843 21791581

[B57] Zschiedrich C. P. Keidel V. Szurmant H. (2016). Molecular mechanisms of two-component signal transduction. J. Mol. Biol. 428, 3752–3775. doi: 10.1016/j.jmb.2016.08.003 27519796 PMC5023499

