# Supplementary material for: Salt stress alters the selectivity of mature pecan for the rhizosphere community and its associated functional traits
Source: Front Plant Sci. 2025 Mar 26;16:1473473. doi: 10.3389/fpls.2025.1473473 (PMC11979281; doi:10.3389/fpls.2025.1473473)
Supplement: Supplementary Table 1 — Sample distribution. [file Table1.doc]

Table S1 samples collection


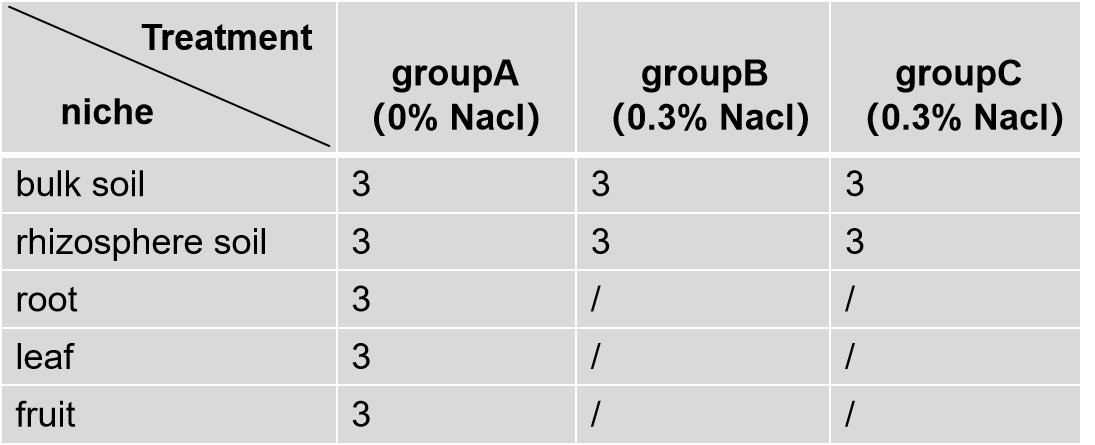


In total, 27 samples were obtained, in which 15 samples from group A including bulk soil , rhizosphere soil, root, leave, and fruit with n=3 for each niche; 12 samples from group B and C including bulk soil and rhizosphere soil with n=3 for each niche.
