# Supplementary figures and images for: Salt stress alters the selectivity of mature pecan for the rhizosphere community and its associated functional traits
Source: Front Plant Sci. 2025 Mar 26;16:1473473. doi: 10.3389/fpls.2025.1473473 (PMC11979281; doi:10.3389/fpls.2025.1473473)

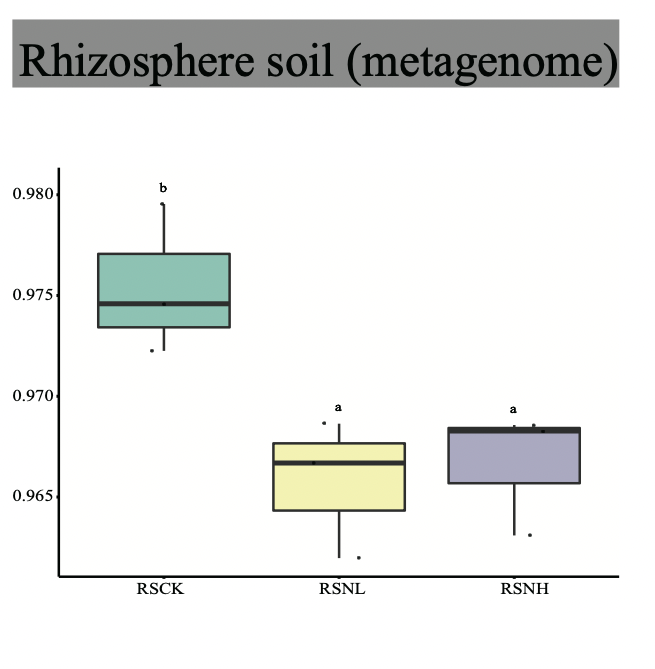

Supplement: Supplementary Figure 1 — Alpha diversity of the rhizosphere soil under nonsalt, low-salt and high-salt treated conditions for bacterial communities by using the metagenonic sequencing data. Box plots show high, low, and median values, with the lower and upper edges of each box denoting the first and third quartiles, respectively. RSCK: rhizosphere soil under nonsalt condition; RSNL: rhizosphere soil under low-salt condition; RSNH: rhizosphere soil under high-salt condition. [file Image1.png]
